# Human Body Burden of Heavy Metals and Health Consequences of Pb Exposure in Guiyu, an E-Waste Recycling Town in China

**DOI:** 10.3390/ijerph182312428

**Published:** 2021-11-26

**Authors:** Wenlong Huang, Xiaoling Shi, Kusheng Wu

**Affiliations:** Department of Preventive Medicine, Shantou University Medical College, Shantou 515041, China; 12wlhuang1@stu.edu.cn (W.H.); 20xlshi@stu.edu.cn (X.S.)

**Keywords:** heavy metals (HMs), body burden, Pb exposure, health outcomes, Guiyu

## Abstract

Guiyu accommodates millions of tons of e-waste from overseas and domestic sources each year and is notorious for its e-waste dismantling industry. As a consequence, Guiyu has been described as “the world’s most toxic place” and “junk town”. Informal e-waste recycling activities have caused severe pollution to the local environment and are associated with extensive health problems to the residents. This review provides updated insights on the body burden of heavy metals derived from e-waste and health outcomes resulted from lead (Pb) exposure. The review identified that Guiyu has been highly contaminated by heavy metals, especially Pb. Excessive exposure to Pb has been associated with multi-system and long-term effects in neonates and children, covering nervous, cardiovascular, adaptive immune, and hematologic systems as well as chromosome and DNA damage. Our review indicates strong associations that emphasize the need to develop strong regulations for prevention of exposure and health consequences in Guiyu and similar sites around the world.

## 1. Introduction

The derived waste from electric and electronic equipment (WEEE, or e-waste) is the fastest growing solid waste stream worldwide, and its management is a significant environmental health concern. China is not only one of the largest e-waste producers and consumers but also the most seriously polluted country subjected to previous illegal importation and informal recycling of e-waste [1,2]. It has been estimated that China would generate 28.4 million tones (Mts) of e-waste in 2030 [3].

**Figure 1:**
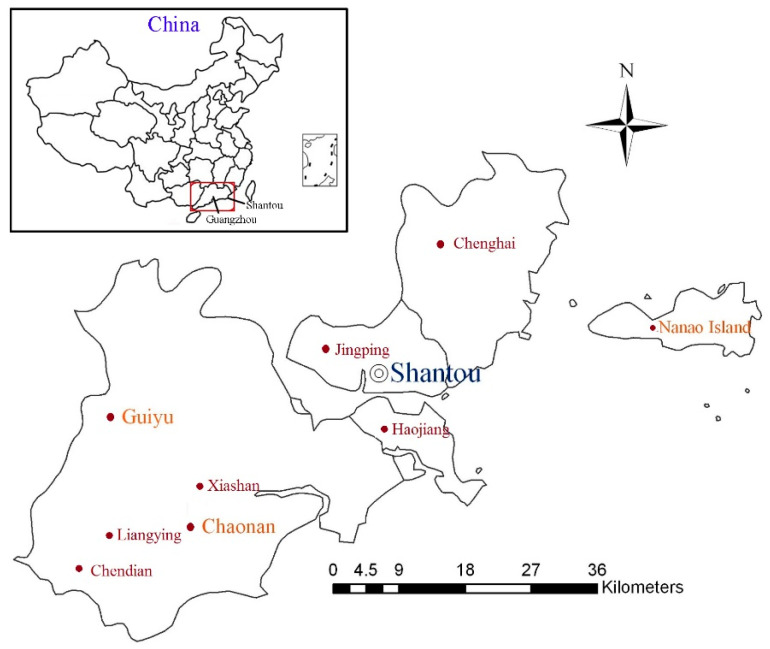
The map of Guiyu and reference areas.

Guiyu town, with a total area of 52.17 km^2^ and a local population of 200,000 (2021), is one of the largest e-waste destinations as a dismantling and recycling center (Figure 1). It accommodates millions of tons of obsolete electronic products from domestic and overseas sources each year and is well known for its e-waste dismantling industry. “The Handbook of Environmental Chemistry” identifies Guiyu as the largest and the second most polluted site in the world [4]. As a consequence, Guiyu has been described as “the world’s most toxic place” or “junk town” [5]. The history of inappropriate shredding and unregulated recycling activities in Guiyu can be traced back to the late 1980s and early 1990s, with a large increase in 1995. 

The long history of recycling and dismantling activities has contributed to the soaring levels of heavy metals (HMs), such as lead (Pb), chromium (Cr), cadmium (Cd), etc., in samples of surrounding air, soil, water, sediment, plants, and dust. HMs enter the human body through three ways: inhalation, dietary intake, and dermal contact. Neonates and children are groups particularly sensitive to HMs because of additional routes of exposure, including placental exposures in uterus, breastfeeding after birth, and high-risk behaviors, such as hand-to-mouth activities in early years and higher risk-taking behaviors in adolescence [6]. Besides, children of e-waste recycling workers also confront take-home contamination from their parents’ clothes and skin. Moreover, they confront direct high-level exposure if their homes are used as family-run disposal and recycling workshops. For these reasons, the environmental burden of HMs has been aggravated [7,8], posing various threats to local residents, especially to vulnerable groups. 

The primary objective of this study was to provide comprehensive information on the body burden and temporal trend of HMs in Guiyu populations, especially children and pregnant women, and to assess the evidence of the association between Pb (the most ubiquitous metal derived from e-waste) and adverse health outcomes.

## 2. Review Methodology

We undertook a systematic search of published literatures using the following inclusion criteria: (i) purpose of the study was to monitor body burden of HMs generated from Guiyu and (ii) explore an association between Pb exposure and outcomes related to physical and mental health. To focus our study, we did not consider other contaminants or studies in other e-waste regions in China. We excluded studies reporting results by in-vitro studies, reviews, or letters to the editor.

We retrieved literatures using the following databases: PubMed, Web of Science, Science Direct, Springer Link, American Chemical Society (ACS Publications), Wiley Online Library, Taylor and Francis Online, CNKI (database of Chinese journal) (http://www.cnki.net/), and Wanfangdata (database of Chinese journal) (https://www.wanfangdata.com.cn) up to May 2021, with the search terms “Guiyu” (“贵屿” in Chinese) as well as the names of HMs (Pb, Cr, Cd, etc.). Our retrieval was not restricted to English; articles published in Chinese were also included.

After preparatory screening, studies deemed relevant were selected for assessment. A total of 73 articles were sure to monitor the body burden of HMs, of which 54 discussed the correlations between Pb exposure and health outcomes.

## 3. Body Burden of HMs in Guiyu

The body burden of HMs from Guiyu includes biological matrices of blood, umbilical cord blood (UCB), placenta, erythrocyte, hair, urine, and meconium.

### 3.1. Total Blood

Many researches have concentrated primarily on blood as a conventional matrix for the surveillance of HMs caused by e-waste exposure; Tables S1–S3 show the HMs level in total blood of Guiyu locals.

**Figure 2:**
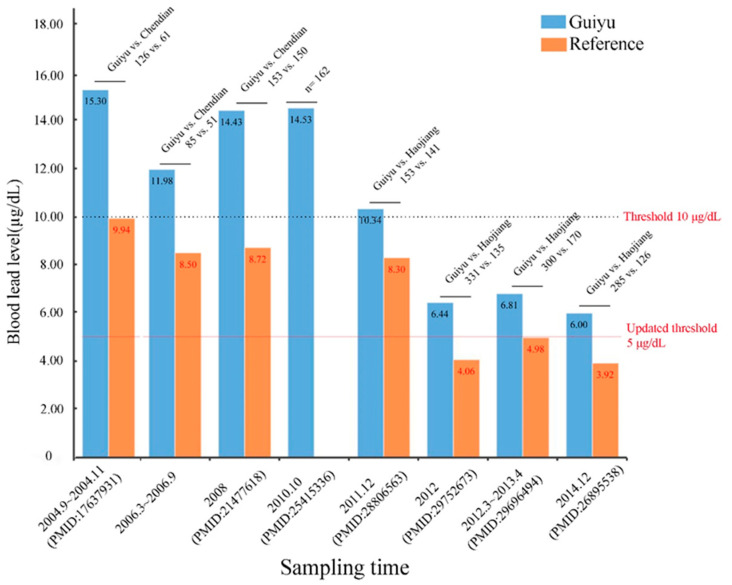
BLLs of children from Guiyu and reference sites from 2004 to 2014; number of children recruited from two groups depicted in each bar (PMID, NCBI PubMed identifier, a standard identifier for an article in PMC).

Pb, one of the ubiquitous and persistent environmental toxicants in e-waste, is associated with many health hazards and has attracted the most attention. Findings from 48 epidemiological studies quantifying blood lead levels (BLLs) of inhabitants living in Guiyu are presented in Table S1. Majority of them (>93%) showed BLLs were higher in Guiyu locals. According to the BLLs diagnostic criteria (10.0 µg/dL) for children defined by the U.S. Centers for Disease Control and Prevention (US-CDC) [9], we found eleven studies [10,11,12,13,14,15,16,17,18,19,20] reporting elevated BLLs. Aside from one study [20] that included no sampling times (used “publication year” as an alternative proxy, see Table S1), all the blood samples of other eight studies were obtained before 2013 (Table S1). There has been a dramatic reduction over the past few years in BLLs of children in Guiyu. This may be attributed to the implementation of restriction policies, such as the *Management Regulation on the Recycling of Waste Electrical and Electronic Products,* promulgated and enforced by the local and national government in the last decade [21,22]. These policies forbid the use of inappropriate and unsafe recycling activities. For example, in 2004, two cross-sectional studies by Xu et al. [19] and Huo et al. [17] found that mean BLLs of children1~6 years old in Guiyu approached 15.30 μg/dL (arithmetic mean). However, in 2014 the BLLs of Guiyu children decreased to 5.06 μg/dL (geometric mean) [23] and 6.00 μg/dL (arithmetic mean) [24]. Elevated BLLs in early childhood are detrimental to neurodevelopment; the recognized adverse effects include impaired cognitive function, attention deficit, hyperactivity, etc. [25]. Following reduction of BLLs observed all over the world, the evaluation threshold of BLL was set lower. In 2012, the CDC updated the threshold value for childhood Pb-exposure downward from 10.0 μg/dL to 5.0 μg/dL [26]. However, there is still no consensus view on how much for Pb exposure is harmful for human health, and it is currently accepted that there is no poisoning safety limit of Pb, particularly for children and neonates. Although BLLs of children in Guiyu have been reduced from around 15 μg/dL to 6.00 μg/dL in the past ten years (Figure 2), the current BLLs of children are still higher than that of reference areas, indicating that children are more sensitive to e-waste exposure and thus have higher potential health effects compared with adults.

Cd is a non-essential element for humans, with high toxicity potential and carcinogenic characteristic; it is toxic to a wide range of organisms and a threat to human health. Hence, the body burden of Cd from Guiyu is a matter of public concern. Findings from 18 epidemiological studies investigating blood Cd levels (BCLs) are shown in Table S2. There were large differences among BCLs, for example, 0.69 μg/L (geometric mean) in 2009~2011 [27], 2.39 μg/L (arithmetic mean) in 2011 [14], 0.12 μg/L (geometric mean) in 2012 [28], and 0.58 μg/L (geometric mean) in 2013 [29]. It seemed that there did not exist a reduction trend for BCLs in children over the past decades. We identified 12 studies comparing the BCLs in Guiyu and reference sites, nine of which showed that children in Guiyu had higher BCLs (Table S2). Nevertheless, for higher BCLs in control areas, Zeng et al. [21] explained that it was somewhat due to dust exposure from fly ash pollution of a thermal power plant in Haojiang. In addition, Zeng et al. [29] found that preserved eggs intake was also a hazardous factor for BCLs elevated. For Cd levels in Guiyu, most children had BCLs > 0.2 μg/L (the current suggested toxicity threshold of Cd).

**Figure 3:**
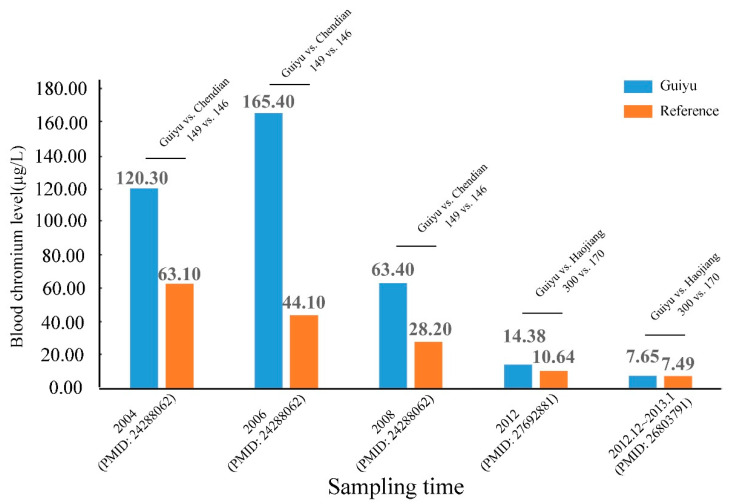
Blood Cr levels of children from Guiyu and reference sites from 2004 to 2012.

Cr is used in metal coatings of some electronic devices for corrosion protection. Cr has two main valence states: Cr (trivalent Cr(Cr ^3+^)) is an essential trace element required for glucose and insulin homeostasis, while Cr (hexavalent Cr (Cr ^6+^)) is known for its genotoxic activity. Body burden of Cr from e-waste has become a worldwide issue of significant public health importance; Guiyu is one of the hardest-hit regions in terms of Cr pollution. Seven studies analyzed the relationships between Cr levels and e-waste exposure (Table S3). Similarly, there has been a dramatic reduction in Cr concentrations of children in Guiyu over the past eight years, ranging from 174.3 μg/L (arithmetic mean) [30] or 165.4 μg/L in 2006 [31] to 7.65 μg/L (geometric mean) in 2012 [28] (Figure 3). However, the normal value of Cr varies person to person; generally, the total quantity of Cr (Cr ^3+^) in an adult body is 5~100 mg. As Cr is distributed in organs, tissues, and body fluid, the normal value of blood Cr is 0~0.2 μg/L, while Cr in tissue is 10~100 times than that in blood [32]. According to Nomiyama et al. [33], normal safety range of blood Cr is 1.0~3.0 μg/L. Zeng et al. [34] found approximately 96% of children in Guiyu had a blood Cr > 5.0 μg/L, numerically higher than control group. Consistent with this study, Zheng et al. [35] demonstrated that blood Cr was slightly higher in children (10~11 years) from Guiyu. Another two studies [28,31] posed the conformity results; one [31] reported that Guiyu children had significantly higher blood Cr in 2004, 2006, and 2008; and the other [28] demonstrated blood Cr was still higher in 2012 (see Table S3 and Figure 3).

Seven studies measured the concentration of manganese (Mn) in Guiyu locals, but results were not consistent and conclusive (Table S3). Lin et al. [28] and Zheng et al. [35] found Mn in children aged 3~7 years and 8~13 years was significantly higher in Guiyu. However, Zeng et al. [21,34] indicated that there was no significant difference of Mn between Guiyu and control areas. Kim et al. [36] found Mn in maternal blood was significantly higher in control area. Other metals (Hg, nickel (Ni), copper (Cu), Se, arsenic (As), zinc (Zn)) are also presented in Table S3.

### 3.2. Umbilical Cord Blood (UCB)

UCB was employed as an indicator to assess potential exposure of embryos and infants. We retrieved nine studies investigated HMs levels in UCB (UCB-HMs) due to e-waste exposure (Table S4). However, the results do not all seem consistent and conclusive. For UCB-Cr, Li et al. [37] found significant differences between neonates in Guiyu and the non-polluted town (2006: 93.87 µg/L vs. 18.10 µg/L; 2007: 70.60 µg/L vs. 24.00 µg/L), whereas no significant difference in another three studies [36,38,39]. In six e-waste Recycling Exposure and Community Health (e-REACH) studies [36,38,39,40,41,42], the UCB-Pb levels from Guiyu were significantly higher than those from five reference areas (Xiamen, Chaonan, Chendian, Shantou, and Haojiang). Four e-REACH studies observed body burden of Cd in umbilical cord (UCB-Cd [36,38,39,43]. According to Ni et al. [38], UCB-Cd levels were significantly higher in Guiyu neonates. Results were consistent with Li et al [43], which indicated that the median UCB-Cd in 2006 and 2007 (but not 2004/2005) were higher in Guiyu neonates. Furthermore, 25.61% of Guiyu newborns exhibited a median UCB-Cd that exceeded the safety limit reported by WHO (5 μg/L), while this rate was 14.18% in control neonates. Within the same study, they observed decreasing concentrations of UCB-Cd from 5.86 μg/L in 2004/2005 to 3.47 μg/L in 2007. However, Kim et al. [36] reported UCB-Cd was significantly higher in Haojiang (0.23 μg/L) than in Guiyu (0.18 μg/L, geometric mean) in 2011 (*p* < 0.05, Table S4), which may be due to the decline of informal e-waste recycling activities in Guiyu from 2004 to 2011. One study [44] investigated UCB-mercury (UCB-Hg) and found that Hg were elevated in Guiyu newborn. Findings from one ecological research [36] revealed that Mn was 1.11-fold higher in Guiyu than in Haojiang. These studies indicated that newborns from Guiyu had a serious body burden of HMs during pregnancy (Table S4).

### 3.3. Placenta

The human placenta, which provides a connection between mother and fetal circulation, mediates transport of nutrients and exchange of gases, eliminates waste, and is critically important for fetal development [45]. Placenta serves as a barrier to the transfer of contaminants to the fetus. However, it has been reported that HMs are able to transfer via the placenta to some degree and penetrate into the developing fetal circulation and may therefore seriously threaten to neonates’ health [46]. Besides, placenta has many advantages for the human species, such as being discarded after the newborn is delivered, and it is easy to obtain and may furnish information on the exposure of both mother and fetus. As a result, placenta is an important non-invasive indicator organ of different organic and inorganic pollution monitoring [47,48]. Nine studies [43,49,50,51,52,53,54,55,56] examined HMs in placentas collected after childbirth (Table S5). As for concentrations of Cd in placenta [43,49,51,52,53,54,55,56], the Cd concentrations in placentas (PCCd) from Guiyu were significantly higher. Inconsistent with the above results, Guo et al. [50] found there was no difference of PCCd between Guiyu and Chaonan. As for Pb, we found six e-REACH studies [49,50,51,54,55,56] that monitored it in placenta (PCPb). According to Guo et al. [50], PCPb was significantly higher in Guiyu in comparison to control area (301.43 ng/g vs. 165.82 ng/g); similar results were found in Xu et al. [56]. However, no difference existed for PCPb in another four studies [49,51,54,55] between Guiyu and reference areas.

### 3.4. Erythrocyte

Three studies [57,58,59] investigated the connection between erythrocyte Pb (EPb) and e-waste exposure (Table S6). They showed that EPb concentration of Guiyu children were higher than that of reference sites. The authors suggested that it would be better to evaluate the Pb toxicity to erythrocytes if combined with blood and erythrocyte Pb level.

### 3.5. Urine

In blood, the half-life of some HMs is short (3~4 months for blood Pb and Cd). However, the half-life of some HMs in other tissues or organs is extremely long. For example, the half-life of Pb in bone reaches 30 years [60]. Cd, a typical nephrotoxicant, is efficiently retained mainly in the kidneys, with a half-life of 10~30 years [61]. It is reported that the concentration of Cd retained in kidney is proportional to that in urine. Therefore, blood Cd can reflect more recent exposure, while Cd excreted in urine is a well-documented biomonitor for assessing lifetime body burden. Four cross-sectional studies [36,62,63,64] assessed the body burden of urine Cd due to e-waste exposure (Table S6). Zhang et al. [63] investigated the impacts of maternal urine Cd in 237 subjects from a Cd-contaminated site and 212 subjects from control, and the levels of urinary Cd were pronouncedly higher among mothers from Guiyu. Similar results could be found in another study [36], while no difference was existed for creatinine-adjusted Cd in children aged 3–7 years [62,64].

### 3.6. Hair

Hair presents many advantages for biomonitoring; for example, it is collected through non-invasive manner, easily transported and stored, and has low cost of acquisition. Therefore, it has been identified as a suitable bio-indicator of monitoring HMs in large cohorts [65]. However, it is worth noting that contamination deposition on hair is more likely to reflect short-term exposure. In this review, we identified two studies [66,67] that investigated HMs (Hg, antimony (Sb)) in engaged workers’ hair to understand the health burden related to e-waste exposure (Table S6). Ni et al. [66] found hair Hg in Guiyu locals were potentially higher than those of Jinping district. Huang et al. [67] demonstrated that Sb in hair of residents from Guiyu were 4.4 times higher than those from Jinping. The existing studies showed a good correlation between Hg/Sb levels and locals’ exposure, suggesting that human hair can be an useful bio-monitor for assessing the extent of toxic metals exposure to locals in areas related to primitive e-waste processing activities.

### 3.7. Meconium

Meconium, the first stools passed by a newborn, is formed mainly from digestive juice secreted by fetal alimentary canal, eliminated mucosa epithelia from neonatal intestinal tract, and other lipids swallowed with amniotic fluid [41]. Meconium provides an ideal matrix, such as ease in sampling and non-invasive analysis. Therefore, meconium acts as a promising bio-monitoring strategy for providing an overview of exposure primarily during the last trimester of pregnancy. However, using meconium as an indicator to assess body burden of HMs in Guiyu’s newborns is lacking. Li et al. [41,68] examined the correlation between meconium Pb (MPb) levels in neonates, indicating Guiyu neonates had significantly higher MPb. Moreover, Li et al. [41] found that there was a correlation between MPb and Pb exposure history during pregnancy.

## 4. Health Effects of Pb Exposure in Guiyu

This section discusses the correlations between Pb exposure and health effects, mainly focusing on neonate and children. Pb can damage a number of systems, including nervous system, cardiovascular system, immune system, hematologic system, and respiratory system (Table S7).

### 4.1. Nervous System

Exposure to environmental pollutants can cause developmental neurotoxicity. Pb, as one of the main neurotoxins, is mainly accumulated in body bone [69]. Neurodevelopmental deficits of children from Pb exposure have aroused great concern. Twelve studies [11,15,16,18,27,41,62,64,70,71,72,73] have examined the effects of exposure, indicating that Pb was associated with irreversible effects on Guiyu residents’ nervous systems, particularly on the developing nervous systems of neonates and children (Table S7). Li et al [41] examined the correlation between Pb and neurobehavior evaluated by neonatal behavioral neurological assessment (NBNA), and found that increased Pb level was associated with low NBNA scores. Moreover, a negative correlation was found between MPb and total NBNA, activity tone, and behavioral scores. Zhang and colleagues [70] indicated that developmental exposure to high levels of Pb resulted in an increase in serum brain-derived neurotrophic factor (BDNF) level and a decrease in child olfactory memory. In another cross-sectional study [11], investigators assessed temperament in children related to Pb exposure, and the mean scores of activity, approach-withdrawal, and adaptability were significantly different between Guiyu and Chendian. Three independent studies [27,71,72] investigated the attention deficit/hyperactivity disorder (ADHD) status among preschool-aged children. Liu et al. [27] found that Pb was correlated with adverse behaviors, including conduct problems, impulsivity-hyperactivity, ADHD index, and Rutter antisocial behavior. Furthermore, serum S100 calcium-binding protein β (S100β) was associated with HMs levels in blood and certain behavioral abnormalities [27]. A later study [72] confirmed that children with high level of Pb (≥10 μg/dL) had 2.4 times higher risk of ADHD than those with low Pb (<10 μg/dL). Moreover, inattentive, hyperactive/impulsive and total scores showed strong positive correlations with Pb, consistent with study by Chen et al. [71]. Han et al. [18] reported that the mean intelligence quotient (IQ) in Guiyu children was lower than that in the reference area (3–4 years) (10.24 vs.12.92, *p* < 0.05). Liu et al. [27] also found that the mean IQ score of children in Guiyu was 95.4, numerically lower than the normative children’s IQ score (103.4) at the same age. Inconsistent with the above results, Wang et al. [74] evaluated the effects of Pb on IQ in children (11–12 years) in Luqiao (an e-waste recycling region), Chun’an (a tinfoil manufacturing area), and Lanxi (a reference town), without differences of IQ among these sites. Liu et al. [62] and Xu et al. [64] assessed the associations of Pb and pediatric hearing ability, and results showed that children had a higher prevalence of hearing loss and higher hearing thresholds (one or both ears) in exposure group. These two studies suggested that early-life exposure to Pb may be an increased risk of hearing impairment; and auditory nervous system of locals might be affected in Pb-polluted areas.

### 4.2. Cardiovascular System

Many studies have delineated a link between early life Pb exposure and blood pressure dysregulation, endothelial dysfunction, lipid metabolism disorders, and subsequent development of cardiovascular disease (CVD) [75,76]. Findings from three epidemiological studies [77,78,79] showed associations between Pb and the cardiovascular system. Lu et al. [78] discovered that Pb impaired vascular structure and aggravated endothelial inflammation, perturbed blood pressure, as well as lowered the ability of lipids clearance, ultimately raising the risk of atherosclerosis. Zheng et al. [77] demonstrated elevated biomarkers of endothelial inflammation (S100A8/A9) and other inflammatory cytokines (such as interleukin-6 (IL-6), interleukin12p70 (IL-12p70), and interferon-inducible protein-10 (IP-10)) of children in Guiyu, indicating that Pb exposure originating from the Pb-containing region may exacerbate endothelial inflammation and contributing to the development of CVD. Chen et al. [79] found that Pb-exposed children manifested smaller left ventricle (including interventricular septum, left ventricle posterior wall, and left ventricle mass index) and impaired left ventricle systolic function (including left ventricle fractional shortening and left ventricle ejection fraction). These studies provide a scientific basis for further in-depth research concerning the effect of Pb on cardiovascular system development.

### 4.3. Immune System

Pb exposure inducing the alterations in immune system has been a matter of increasing scientific and public concern. Twelve studies [14,23,24,28,57,59,80,81,82,83,84] have examined the effects of Pb on immune functions (Table S7). Quantification of vaccine-induced antibodies, including MMR (measles, mumps, rubella) antibody (Ab) [80], hepatitis B surface Ab (HBsAb) [81], diphtheria Ab, pertussis Ab, tetanus Ab, Japanese encephalitis Ab, polio Ab, and measles Ab [28], showed children from Guiyu were significantly lower, suggesting a reduced immunity of children living in Pb-exposed area. Xu et al. [81] demonstrated that HBsAb levels were negatively associated with Pb in Guiyu children. One later study [28] found hepatitis B antibody level was negatively correlated with Pb. Zhang et al. [24] evaluated the Pb-exposure effects on natural killer (NK) cells and NK cell activation-associated cytokine/chemokine and found that the percentages of NK cell and concentrations of cytokines and chemokines (interleukin-2 (IL-2), interleukin-27 (IL-27), macrophage inflammatory protein-1a (MIP-1a), and MIP-1b) were remarkedly lower in Guiyu children. A similar study by Cao et al. [23] indicated that children in Guiyu harbored higher percentages of CD4^+^ and CD8^+^ central memory T cells but not for cytokines (IL-2, interleukin-7 (IL-7), interleukin-15 (IL-15)). Dai et al. [57] evaluated effect of Pb on erythrocyte complement receptor 1 (CR1/CD35) expression and immunologic function of preschool children and indicated that elevated Pb levels resulted in adverse changes in CR expression, suggesting that Pb might be a non-negligible threat to erythrocyte immunity development. Zheng et al. focused on the adverse effects of Pb on IgG subclass production and found increased Pb level was accompanied by lower serum levels of IL-13 and higher levels of IFN-γ, IgG1, and IgG1 + IgG2 in exposed children. All these findings indicate that Pb exposure results in alterations in immune function of children.

### 4.4. Hematologic System

Pb exposure may lead to deleterious effect on hematologic system. Eleven studies investigated the adverse effects of Pb on the hematologic system (Table S7), six [29,57,58,85,86,87] of which were related to effects on hemoglobin (Hgb) synthesis and three [24,29,88] of which assessed the effects of Pb on the status of peripheral blood platelet. Zeng et al. [29,88] aimed to look at the effects of Pb on platelet indices (including platelet count (PLT), plateletcrit (PCT), platelet distribution width (PDW), mean platelet volume (MPV), and platelet large cell ratio (P-LCR)) in preschool children from Guiyu. Both found PCT were conspicuously higher in Guiyu children aged 5–7 years and 3–7 years, respectively. In addition, Zeng et al. [88] also found significant relationships between Pb and PLT, PCT, MPV, and P-LCR. Moreover, both studies found the median PLT and PCT levels of children residence in Guiyu exceeded the recommended maximum reference range value 300 × 10^9^/L and 0.3%, respectively. However, the reported effects of Pb on Hgb are not consistent, with two studies [57,58] reporting Hgb of children aged 2–6 and 3–7 years was numerically higher (both *p* > 0.05), one [29] reporting Hgb of 5–7 year children was significantly lower in Guiyu (*p* < 0.05), and one [85] demonstrating that Hgb of hospitalized patients aged 4–85 years from Guiyu was significantly higher. Moreover, the former three studies and Wang et al. [87] demonstrated that Pb was negatively associated with Hgb in preschool children; the latter found Pb positively correlated with Hgb. The above results indicate Pb’s hematotoxicity on inhibiting hemoglobin synthesis and/or metabolism, yet the mechanisms of action of Pb on hemoglobin still need to be elucidated.

### 4.5. Growth Effects

Pb is associated with physical growth and development of children by inhibiting ions (calcium (Ca^2+^), iron (Fe^2+^), and other metal ions) uptake and blocking synthesis and utilization of hormone [89]. We identified 27 studies that analyzed the associations between Pb and physical growth (Table S7). The reported effects of Pb exposure on child physical growth indicators, such as body weight, height, and BMI, were not completely consistent. Of these, twelve studies showed that height, weight, body mass index (BMI), and chest or head circumference were significantly lower in children living in Guiyu. Nevertheless, five studies [12,15,16,78,84] revealed that BMI was significantly higher in Guiyu children. In addition, height [34,90], weight [13,34,90], BMI [34], head circumference [34], and chest circumference [34] were reported negatively correlated with Pb. Moreover, Pb was positively associated with bone resorption biomarkers (including serum calcium, osteocalcin, bone alkaline phosphatase, and urinary deoxypyridinoline) among children aged 3–8, indicating that Pb exposure may have detrimental consequences on child physical growth and development [90]. Taken together, living in a Pb-contaminated area would have long-term effect on growth and development of children; worse still, these harmful effects may persist into their adolescence, greatly diminish life quality, and increase social burden.

### 4.6. Adverse Birth Outcomes

Fifteen studies investigated the association between Pb and adverse birth outcomes (Table S7). Xu et al. [40] described birth outcomes and levels of UCB-Pb among neonates from Guiyu in comparison to those from Xiamen (from 2001 to 2008). In detail, the study reported that Guiyu births had significantly higher rates of adverse birth outcomes (such as stillbirth: 4.72% vs. 1.03%; lower birth weight: 6.12% vs. 4.12%; lower Apgar scores: 9.6 vs. 9.9) than did births from Xiamen. More studies have come to similar results (Table S7).

### 4.7. Chromosome and DNA Damage

People dwelling in an e-waste town or working related to e-waste had evidence of greater DNA damage. We identified six studies that evaluated adverse effects at cellular and molecular level caused by Pb exposure (Table S7). Li et al. [37] and Wang et al. [91] observed significant differences in lymphocytic DNA damage in neonates between Guiyu and references. They found that DNA damage of cord blood cells in injury rate and lengths of tail in the comet assay were significantly higher of Guiyu neonates. Ni et al. [38] evaluated the effects of Pb on DNA oxidative damage in neonates by using 8-hydroxydeoxyguanosine (8-OHdG) in UCB as a biomarker. However, no significant association of 8-OHdG and Pb was found. Xu et al. [92] investigated the influence of Pb on reactive oxygen species (ROS)-induced DNA damage; they found that high Pb exposure had significantly higher urinary 8-OHdG than low exposure. Another study explored associations between DNA methylation patterns in newborns and maternal Pb exposure during pregnancy and highlighted the epigenetic-modified changes of the fetus under maternal Pb exposure [39]. These studies demonstrate the cytotoxic and genotoxic effects of Pb and its potential mutagenicity, which might be the molecular mechanisms for Pb-induced health effects.

## 5. Conclusions

This report systematically reviewed studies to assess the evidence of body burden of HMs in Guiyu neonate and children (pregnant mother also) as well as health concerns arising from Pb exposure. Excessive exposure to Pb has been associated with multi-system and long-term effects. In the past decades, informal waste processing and disposal has been rampant throughout this area, causing serious environmental contamination and posing various threats to residents’ health. Although e-waste management has become a great concern in the last few years, and the local government has implemented some measures and policies to reduce primitive dismantling activities, the rigorous implementation of regulations is an ongoing challenge. E-waste is not just an environmental problem of one region or one country; it is a worldwide universal issue. The hazardous effects of HMs derived from e-waste must be of precedent concern of the public in order to provide more evidence of the body load and the health effects of toxic metals.

## Data Availability

The data presented in this study are available on request from the corresponding author.

## Supplementary Materials

The following are available online at https://www.mdpi.com/article/10.3390/ijerph182312428/s1, Table S1: Pb in blood (µg/dL); Table S2: Cd in blood (µg/L); Table S3: Other metals (Cr, Mn, Ni, Hg, As, Cu, Zn, and Se) in blood; Table S4: Metals in umbilical cord blood (UCB); Table S5: Metals in placenta; Table S6: Metals in other human matrix (urine, meconium, erythrocyte, and hair); Table S7: The effects of Pb exposure on several health outcomes in neonate, children, and adults in Guiyu.
