# Supplementary material for: Human Body Burden of Heavy Metals and Health Consequences of Pb Exposure in Guiyu, an E-Waste Recycling Town in China
Source: Int J Environ Res Public Health. 2021 Nov 26;18(23):12428. doi: 10.3390/ijerph182312428 (PMC8657058; doi:10.3390/ijerph182312428)
Supplement: Supplementary file 1 [file ijerph-18-12428-s001.zip › ijerph-1441855-supplementary.pdf]

Supplementary Material

Human body burden of heavy metals and health consequences of Pb exposure in Guiyu, an e-waste recycling town in China: A critical review

Wenlong Huang, Xiaoling Shi and Kusheng Wu *

Department of Preventive Medicine, Shantou University Medical College, Shantou 515041, Guangdong, China

* Corresponding author:
Kusheng Wu, Ph.D.
Department of Preventive Medicine, Shantou University Medical College
No.22, Xinling Rd. Shantou 515041, Guangdong, China
Tel: 86-754-88900445
Fax: 86-754-88557562
E-mail: kswu@stu.edu.cn

This file includes:

1. Tables S1 to S6: Body burden of HMs in Guiyu
2. Tables S7: Correlations between Pb exposure and health outcomes
3. References

Table S1 Pb in blood (μg/dL)

Ref. No.	Sampling time	Age	Guiyu	Reference areas	*p* value	Data genre	Reference
1	2004	< 6 years	15.30	9.94	< 0.01	Mean	(Huo et al., 2007)
2	2004.9~2004.11	1~6 years	15.30	9.94	< 0.01	Mean	(Xu et al., 2006)
3	2005*	1~6 years	15.30	9.94	< 0.01	Mean	(Peng et al., 2005)
4	2006.3~2006.9	3~6 years	11.98	8.50	< 0.01	Mean	(Han et al., 2007)
5	2006.5~2006.6	1~7 years	13.17	10.04	< 0.01	Mean	(Zheng et al., 2008)
6	2008.1~2008.2	3~7 years	14.43	8.72	< 0.01	Mean	(Liu et al., 2011a)
7	2010.10	4~6 years	14.53			Mean	(Xu et al., 2014)
8	2011*	3~7 years	8.20			Mean	(Liao et al., 2011)
9	2011.12	3~7 years	10.34	8.30	< 0.001	Mean	(Zhang et al., 2017b)
10	2012	3~7 years	6.44	4.06	< 0.01	Mean	(Zeng et al., 2018)
11	2012.3~2013.4	Preschool	6.81	4.98	< 0.001	Mean	(Zeng et al., 2019a)
12	2014.12	3~7 years	6.00	3.92	< 0.001	Mean	(Zhang et al., 2016)
13	2017*	4~7 years	9.40	5.04	< 0.001	Mean	(Zhang et al., 2017a)
14	2006~2011	3~7 years	8.19	6.72	< 0.01	Median	(Liu et al., 2015a)
15	2008	3~7 years	13.89	8.55	< 0.01	Median	(Xu et al., 2015c)
16	2009~2011	3~7 years	7.33			Median	(Liu et al., 2014)
17	2011.9~2012.8	Pregnant women	6.66	3.81	< 0.05	Median	(Kim et al., 2019)
18	2011~2013	18~24 years	6.3	3.5			(Kim et al., 2020)
		25~29 years	6.8	3.8			
		>30 years	6.9	4			
19	2011.12~2012.3	3 years	11.30	5.77	< 0.001	Median	(Liu et al., 2015b)
20	2011.12~2012.3	3 years	11.30	5.77	< 0.001	Median	(Liu et al., 2018a)
21	2011.12~2012.3	< 11 years	7.06	5.89	< 0.05	Median	(Guo et al., 2014)
22	2011.12~2012.3	3~7 years	6.76	6.05	0.0020	Median	(Xu et al., 2015a)
	2011	3~7 years	8.76	7.89	0.0310	Median	
	2012	3~7 years	5.83	4.61	< 0.001	Median	
23	2012.1~2012.5	3~7 years	7.90			Median	(Zhang et al., 2015)
24	2012	3~7 years	9.43	6.79	< 0.001	Median	(Lin et al., 2017b)
25	2012*	3~6 years	9.64	7.84	< 0.001	Median	(Sun et al., 2012)
26	2012.12~2013.1	3~8 years	6.24	4.75	< 0.001	Median	(Zeng et al., 2016)
27	2013*	3~8 years	7.30			Median	(Yang et al., 2013)
28	2013*	2~8 years	7.89			Median	(Chen et al., 2013)
29	2013.11~2013.12	5~7 years	5.53	3.57	< 0.001	Median	(Zeng et al., 2017)
30	2013.12	Preschool	5.70	3.53	< 0.01	Median	(Zheng et al., 2021)
31	2014	3~7 years	5.06	3.60	< 0.001	Median	(Cao et al., 2018)
32	2014	3~6 years	8.50	6.00	< 0.001	Median	(Wang et al., 2021)
33	2014	3~7 years	4.94	3.85	< 0.001	Median	(Liu et al., 2018b)
34	2014.12	3~7 years	3.72	2.27	< 0.001	Median	(Zhang et al., 2020b)
35	2014.12	3~7 years	5.29	3.63	< 0.001	Median	(Xu et al., 2020)

36	2014.12~2015.3	2~7 years	5.61	3.57	< 0.001	Median	(Lin et al., 2016)
37	2014.1~2016.12	44~80 years	2.93 ~ 4.06	3.14 ~ 3.79	> 0.05	Median	(Lin et al., 2017a)
38	2014.12~2015.6	4~85 years	8.70	5.10	0.0010	Median	(Chen et al., 2019)
39	2015.11	2~6 years	6.50	4.50	< 0.01	Median	(Dai et al., 2017)
40	2015.11~2015.12	2~7 years	6.51	4.41	< 0.01	Median	(Huo et al., 2019)
41	2016.11~2016.12	3~7 years	7.14	3.91	< 0.001	Median	(Lu et al., 2018)
42	2016.11~2016.12	3~7 years	7.23	3.91	< 0.001	Median	(Zheng et al., 2019)
43	2017	3~7 years	4.89	3.47	< 0.001	Median	(Zhang et al., 2020a)
44	2017.10~2017.12	3~7 years	5.19	3.42	< 0.001	Median	(Zeng et al., 2021)
45	2017.11~2017.12	3~6 years	4.88	3.47	< 0.001	Median	(Cai et al., 2019)
46	2017.11~2017.12	2.5~6 years	4.86	3.47	< 0.001	Median	(Hou et al., 2020)
47	2018*	3~6 years	7.43			Median	(Xu et al., 2018)
48	2018.11~2018.12	2~7 years	4.51	3.98	< 0.001	Median	(Chen et al., 2021)

*Year of article published (As no "sampling time" provided in some original publications, we used "publication year" as an alternative proxy, similarly hereinafter)

Table S2 Cd in blood (μg/L)

Ref. No.	Sampling time	Age	Guiyu	Reference areas	P value	Data genre	Reference
1	2006.5~2006.6	1~7 years	1.58	0.97	< 0.01	Mean	(Zheng et al., 2008)
2	2010.10	4~6 years	0.77			Mean	(Xu et al., 2014)
3	2011.12	3~7 years	2.39	1.79	< 0.001	Mean	(Zhang et al., 2017b)
4	2004.5~2009.10	3~7 years				Median	(Lin et al., 2010)
5	2009~2011	3~7 years	0.69			Median	(Liu et al., 2014)
6	2011.9~2012.8	Pregnant women	1.72	1.43	< 0.05	Median	(Kim et al., 2019)
7	2011.12~2012.3	3 years	1.22	0.72	< 0.001	Median	(Liu et al., 2018a)
8	2012.1~2012.5	3~7 years	0.95			Median	(Zhang et al., 2015)
9	2012	3~7 years	0.12	0.27	< 0.001	Median	(Lin et al., 2017b)
10	2012*	3~6 years	2.29	1.68	< 0.001	Median	(Sun et al., 2012)
11	2012.3~2013.4	Preschool	0.66	0.54	< 0.001	Median	(Zeng et al., 2019a)
12	2012.12~2013.1	3~8 years	0.58	0.50	< 0.01	Median	(Zeng et al., 2016)
13	2013*	3~8 years	0.69			Median	(Yang et al., 2013)
14	2013.11~2013.12	5~7 years	0.58	0.57	0.7170	Median	(Zeng et al., 2017)
15	2013.12	Preschool	0.56	0.47	< 0.05	Median	(Zheng et al., 2021)
16	2014.12	3~7 years	0.33	0.23	< 0.001	Median	(Zhang et al., 2020b)
17	2015.12~2015.6	4~85 years	2.10	2.60	0.4600	Median	(Chen et al., 2019)
18	2018*	3~6 years	0.72			Median	(Xu et al., 2018)

*Year of article published

Table S3 Other metals (Cr, Mn, Ni, Hg, As, Cu, Zn and Se) in blood

Ref. No.	Sampling time	Age	Guiyu	Reference areas	*p* value	Data genre	Reference
**Cr in Blood (µg/L)**							
1	2004	3~7 years	120.30	63.10	< 0.0001	Mean	(Xu et al., 2015d)
	2006	3~7 years	165.40	44.10	< 0.0001	Mean	
	2008	3~7 years	63.40	28.20	< 0.0001	Mean	
2	2011*	3~7 years	174.30			Mean	(Liu et al., 2011b)
3	2013*	8~13 years	35.50	34.10	> 0.05	Median	(Zheng et al., 2013)
4	2011.9~2012.8	Pregnant women	13.78	8.90	< 0.05	Median	(Kim et al., 2019)
5	2012	3~7 years	14.38	10.64	0.0050	Median	(Lin et al., 2017b)
6	2012.12~2013.1	3~8 years	7.65	7.49	0.3790	Median	(Zeng et al., 2016)
7	2012.3~2013.4	Preschool			> 0.05		(Zeng et al., 2019a)
**Mn in Blood (µg/L)**							
1	2013*	8~13 years	20.60	14.90	< 0.01	Median	(Zheng et al., 2013)
2	2011.9~2012.8	Pregnant women	25.93	28.51	< 0.05	Median	(Kim et al., 2019)
3	2012	3~7 years	16.31	15.08	0.0300	Median	(Lin et al., 2017b)
4	2012.3~2013.4	Preschool			> 0.05		(Zeng et al., 2019a)
5	2012.12~2013.1	3~8 years	28.18	28.09	0.9330	Median	(Zeng et al., 2016)
6	2009~2011	3~7 years	17.98			Median	(Liu et al., 2014)
7	2014.1~2016.12	44~80 years	12.10 ~ 20.10	9.00 ~ 11.10	< 0.01	Median	(Lin et al., 2017a)
**Ni in Blood (µg/L)**							
1	Not given	8~13 years	5.30	3.00	< 0.01	Median	(Zheng et al., 2013)
2	2012	3~7 years	4.41	3.44	0.0590	Median	(Lin et al., 2017b)
**Hg in Blood (µg/L)**							
1	2012	3~7 years	1.92	2.25	0.0510	Median	(Lin et al., 2017b)
2	2018*	3~6 years	11.13				(Xu et al., 2018)
2	2014.12	3~7 years	1.46	1.10	< 0.001	Median	(Zhang et al., 2020b)
**As in Blood (µg/L)**							
1	2012	3~7 years	5.53	6.56	0.0020	Median	(Lin et al., 2017b)
2	2014.12	3~7 years	5.91	4.18	< 0.001	Median	(Zhang et al., 2020b)
**Cu in Blood (µg/L)**							
1	2012	3~7 years	919.10	842.45	< 0.001	Median	(Lin et al., 2017b)
2	2014.1~2016.12	44~80 years	780.70 ~ 966.40	740.50 ~ 986.30	> 0.05	Median	(Lin et al., 2017a)
**Zn in Blood (µg/L)**							
1	2012	3~7 years	4746.39	4313.03	0.0390	Median	(Lin et al., 2017b)
**Se in Blood (µg/L)**							
1	2012	3~7 years	135.77	149.06	0.0270	Median	(Lin et al., 2017b)

*Year of article published

Table S4 Metals in umbilical cord blood (UCB)

Ref. No.	Sampling time	Guiyu	Reference areas	*p* value	Data genre	References
**Cr in UCB (μg/L)**						
1	2006	93.87	18.10	< 0.01	Median	(Li et al., 2008a)
	2007	70.60	24.00	< 0.01	Median	
2	2011.6~2012.9	5.90	6.23	> 0.05	Mean	(Zeng et al., 2019b)
3	2011.9~2012.8	4.02	3.52	> 0.05	Median	(Kim et al., 2019)
4	2012.3~2013.1	27.52	26.42	> 0.05	Median	(Ni et al., 2014b)
**Pb in UCB (μg/dL)**						
1	2001~2008	11.59	2.33	< 0.01	Mean	(Xu et al., 2012)
2	2006.7~2006.10	11.33	6.04	< 0.01	Mean	(Li et al., 2008c)
3	2011.6~2012.9	7.34	3.07	< 0.001	Mean	(Zeng et al., 2019b)
4	2004.12~2005.6	10.50	7.79	< 0.05	Median	(Huo et al., 2014)
5	2011.9~2012.8	5.03	3.18	< 0.05	Median	(Kim et al., 2019)
6	2012.3~2013.1	11.05	5.73	< 0.001	Median	(Ni et al., 2014b)
**Cd in UCB (μg/L)**						
1	2004~2005	5.86	4.84	> 0.05	Mean	(Li et al., 2011)
	2006	5.30	1.82	< 0.01	Mean	
	2007	3.47	0.94	< 0.01	Mean	
	2004~2007	4.84	2.81	< 0.01	Mean	
2	2011.6~2012.9	0.22	0.29	> 0.05	Mean	(Zeng et al., 2019b)
3	2011.9~2012.8	0.18	0.23	< 0.05	Median	(Kim et al., 2019)
4	2012.3~2013.1	2.50	0.33	< 0.001	Median	(Ni et al., 2014b)
**Hg in UCB (μg/L)**						
1	2008.1~2010.12	8.52	8.37	< 0.01	Median	(Zhuang et al., 2015)
**Ni in UCB (μg/L)**						
1	2012.3~2013.1	8.63	9.09	> 0.05	Median	(Ni et al., 2014b)
**Mn in UCB (μg/L)**						
1	2011.9~2012.8	52.93	49.69	< 0.05	Median	(Kim et al., 2019)
2	2011.6~2012.9	54.01	51.21	> 0.05	Mean	(Zeng et al., 2019b)

Table S5 Metals in placenta

Ref. No.	Sampling time	Guiyu	Reference areas	*p* value	Data genre	Reference
**Cd in Placenta (ng/g wt)**						
1	2006	170.00	100.00	< 0.01	Mean	(Li et al., 2011)
2	2006.5~2006.9	170.00	100.00	< 0.05	Mean	(Li et al., 2010)
3	2012.3~2012.8	96.19	12.65	< 0.001	Mean	(Xu et al., 2016)
4	2006~2010	92.90	23.90	< 0.01	Median	(Lin et al., 2013)
5	2008.10~2009.5	108.75	104.15	0.8420	Median	(Guo et al., 2010)
6	2008.10~2009.6	83.99	51.59	< 0.001	Median	(Zhang et al., 2011)
7	2010.9~2011.9	96.56	20.87	< 0.01	Median	(Xu et al., 2015b)
8	2010.9~2011.9	96.60	20.90	< 0.01	Median	(Qiu et al., 2012)

9	2012*	96.56	21.15	< 0.001	Median	(Qiu, 2012)
**Pb in Placenta (ng/g wt)**						
1	2012.3~2012.8	498.80	27.01	< 0.001	Mean	(Xu et al., 2016)
2	2006~2010	1249.10	1353.50	> 0.05	Median	(Lin et al., 2013)
3	2008.10~2009.5	301.43	165.82	0.0100	Median	(Guo et al., 2010)
4	2008.10~2009.6	521.01	273.24	0.2990	Median	(Zhang et al., 2011)
5	2012*	-	-	> 0.05		(Qiu, 2012)
6	2010.9~2010.12	399.90	421.00	> 0.05	Median	(Qiu et al., 2012)
**Cr in Placenta (ng/g wt)**						
1	2008.10~2009.5	234.31	228.40	0.7360	Median	(Guo et al., 2010)
**Ni in Placenta (ng/g wt)**						
1	2008.10~2009.5	7.64	14.30	< 0.001	Median	(Guo et al., 2010)

*Year of article published

Table S6 Metals in other human matrix (urine, meconium, erythrocyte and hair)

**Ref. No.**	**Sampling time**	**Age**	**Guiyu**	**Reference areas**	***P* value**	**Data genre**	**Reference**
**Pb in erythrocyte (μg/dL)**							
1	2006~2011	3~7 years	21.95	17.32	< 0.01	Median	(Liu et al., 2015a)
2	2015.11	2~6 years	17.00	11.90	< 0.001	Median	(Dai et al., 2017)
3	2015.11~2015.12	2~7 years	16.605	11.771	< 0.001	Median	(Huo et al., 2019)
**Cd in urine (μg/g creatinine)**							
1	2011.9~2012.6	Pregnant women	1.38	0.75	< 0.01	Mean	(Zhang et al., 2018)
			1.59	0.76	< 0.01	Mean	
			0.92	0.67	< 0.01	Median	
			1.00	0.59	< 0.01	Median	
2	2011.9~2012.8	Pregnant women	1.06	0.54	< 0.05	Median	(Kim et al., 2019)
3	2014	3~7 years	2.49	1.80	0.1340	Median	(Liu et al., 2018b)
4	2014.12	3~7 years	1.52	1.21	0.3230	Median	(Xu et al., 2020)
**Cr in urine (μg/g creatinine)**							
1	2011.9~2012.8	Pregnant women	1.66	0.97	< 0.05	Median	(Kim et al., 2019)
**Mn in urine (μg/g creatinine)**							
1	2011.9~2012.8	Pregnant women	7.62	3.14	< 0.05	Median	(Kim et al., 2019)
**Hg in hair (μg/g)**							
1	2012.4~2012.6	Residents	1.12	0.65	< 0.001	Mean	(Ni et al., 2014a)
			0.99	0.59	< 0.001	Median	
**Sb in hair (ng/g)**							
1	2012.4~2012.6	Residents	389.66	87.96	< 0.001	Mean	(Huang et al., 2015)
			160.78	61.74	< 0.001	Median	
**Pb in meconium (μg/g)**							
1	Not given	Neonates	1.93	1.01	< 0.01	Mean	(Li et al., 2008b)
2	2006.7~2006.10	Neonates	2.50	1.20	< 0.01	Mean	(Li et al., 2008c)

Table S7. The effects of Pb exposure on several health outcomes in neonate, children and adults in Guiyu

Reference	Exposed population	Matrix*	Health consequences
<u>**Nervous system**</u>			
(Li et al., 2008c)	Guiyu vs. Chaonan (Neonate)	MPb, UCB-Pb	Negative associations between Pb and total NMNA, activity tone, and behavior scores.
(Zhang et al., 2017a)	Guiyu vs. Haojiang (4~ 7 years, n=118)	BPb	BDNF was positively correlated with Pb. Olfactory memory tests scores negatively correlated with Pb.
(Liu et al., 2015b)	Guiyu vs. Nanao (3 years, n=284)	BPb	Lower cognitive and language scores in Guiyu children (both $p<0.001$).
(Liu et al., 2018a)	Guiyu vs. Nanao (3 years, n=284)	BPb	Pb negatively correlated with cognitive and language scores (both $p<0.001$)
(Liu et al., 2014)	Guiyu only (3~7 years, n=240)	BPb	Lower IQ score (95.4), Pb positively correlated with conduct problems, impulsivity-hyperactivity, ADHD index and Rutter antisocial behavior.
(Zhang et al., 2015)	Guiyu only (3 ~7 years, n=243)	BPb	ADHD scores positively correlated with Pb, children with high Pb (≥10 μg/dL) had 2.4 times higher risk of ADHD than those with low Pb (<10 μg/dL).
(Chen et al., 2013)	Guiyu only (2 ~8 years, n=172)	BPb	Positive correlations among Pb and the total and subtypes scores of ADHD (all $p<0.01$).
(Liu et al., 2011a)	Guiyu vs. Chendian (3~7 years, n=303)	BPb	Higher scores of activity level, approach-withdrawal, and adaptability dimensions in Guiyu children (all p<0.01).
(Han et al., 2007)	Guiyu vs. Chendian (3~6 years, n=136)	BPb	Lower IQ score in Guiyu children (aged 3~4 years) ($p<0.01$)
(Liu et al., 2018b)	Guiyu vs. Haojiang (3 ~7 years, n=234)	BPb	Elevated hearing thresholds and hearing loss prevalence in Guiyu children. Pb showed a significant OR (1.24) for hearing loss in children by adjusting confounders.
(Xu et al., 2020)	Guiyu vs. Haojiang (3 ~7 years, n=116)	BPb	Higher hearing thresholds in either ear of the exposed children

(Cai et al., 2019)	Guiyu vs. Haojiang (3 ~6 years, n=574)	BPb	Sensory processing scores were positively correlated with Pb.
**Cardiovascular system**			
(Lu et al., 2018)	Guiyu vs. Haojiang (3 ~7 years, n=590)	BPb	lnBPb was positively associated with TG (p<0.05) and negatively with HDL (p<0.05), SBP and PP (both p<0.01).
(Zheng et al., 2019)	Guiyu vs. Haojiang (3 ~7 years, n=203)	BPb	Pb was positively related to IL-6, IL-12p70, IP-10.
(Chen et al., 2021)	Guiyu vs. Haojiang (3 ~7 years, n=486)	BPb	Smaller left ventricle and impaired systolic function were found in Pb-exposed children.
**Immune system**			
(Zhang et al., 2016)	Guiyu vs. Haojiang (3 ~7 years, n=411)	BPb	IL-1β and IL-6 were positively associated with Pb. NK cells were negatively correlated with Pb. IL-27 was negatively associated with Pb.
(Zhang et al., 2017b)	Guiyu vs. Haojiang (3 ~7 years, n=294)	BPb	Higher absolute counts of monocytes, eosinophils, neutrophils (all p<0.001) and basophils (p<0.05), higher percentages of eosinophils (p<0.01) and neutrophils in (p<0.001), lower percentages of NK cells (p<0.001) in Guiyu children.
(Dai et al., 2017)	Guiyu vs. Haojiang (2 ~6 years, n=484)	BPb EPb	Both BPb and EPb negatively related to CR1 levels.
(Lin et al., 2016)	Guiyu vs. Haojiang (2 ~7 years, n=378)	BPb	Lower antibody titers against MMR (measles, mumps, and rubella) in Guiyu children (all p<0.001).
(Xu et al., 2015a)	Guiyu vs. Haojiang (3 ~7 years, n=590)	BPb	Lower hepatitis b surface antibody levels (HBs Ab 1.04 vs. 4.06 s/co, p<0.001) in Guiyu children. Pb was negatively associated with HBs Ab.
(Sun et al., 2012)	Guiyu vs. Haojiang (3 ~6 years, n=237)	BPb	Lower percentages of NK cells in Guiyu group.

(Lin et al., 2017b)	Guiyu vs. Haojiang (3 ~7 years, n=284)	BPb	Lower levels of vaccine antibodies (Diphtheria, Pertussis, Tetanus, Japanese encephalitis, Polio, Measles) in Guiyu children (all p<0.01). All vaccine antibodies negatively correlated with Pb.
(Cao et al., 2018)	Guiyu vs. Haojiang (3 ~7 years, n=118)	BPb	Pb was positively associated with CD4+ central memory T cells, negatively associated with CD4+ Tn cells.
(Zheng et al., 2019)	Guiyu vs. Haojiang (3 ~7 years, n=203)	BPb	Higher percentages of $CD4^+$ T cells (56.52% vs. 52.53%, p<0.01) in Guiyu children.
(Huo et al., 2019)	Guiyu vs. Haojiang (2 ~7 years, n=267)	BPb	Higher erythrocyte Pb was associated with lower CD44 and CD58, BPb correlated with higher IL-12p70 and IFN-γ, and lower IL-2
(Zhang et al., 2020b)	Guiyu vs. Haojiang (2 ~7 years, n=147)	BPb	Anti-inflammatory IL-1RA concentration was negatively related with Pb; pro-inflammatory IL-1b and IL-6 were positively correlated with Pb.
(Zheng et al., 2021)	Guiyu vs. Haojiang (2 ~7 years, n=324)	BPb	Pb positively associated with serum levels of IFN-γ, and negatively associated with serum levels of IL-13.
**Hematologic System**			
(Zeng et al., 2018)	Guiyu vs. Haojiang (3 ~7 years, n=466)	BPb	Elevated PLT, PCT, MPV (all p<0.001), and P-LCR (p<0.01) in Guiyu children. Positive correlation between Pb and PLT, PCT, PDW, MPV and P-LCR.
(Liu et al., 2015a)	Guiyu vs. Chendian & Chaonan (3~7 years, n=855)	BPb, EPb	Hgb and HCT negatively correlated with EPb (both p<0.001). EPb is better than BPb to assess hematoxicity of Pb. EPb is negatively associated with Hgb.
(Zhang et al., 2016)	Guiyu vs. Haojiang (3 ~7 years, n=411)	BPb	Elevated PLT (p<0.05), decrease lymphocyte (p<0.01), no significant differences in monocyte, erythrocyte, neutrophil. PLT, neutrophil and monocyte counts correlated with Pb.
(Zeng et al., 2017)	Guiyu vs. Haojiang & Xiashan (5 ~7 years, n=206)	BPb	Lower Hgb (128.17 vs. 131.49 g/L, p<0.05). Elevated PLT, PCT, RDW-SD and RDW-CV (all p<0.001). Living in Guiyu was positively associated with PLT (p<0.01), negatively associated with Hgb.

(Dai et al., 2017)	Guiyu vs. Haojiang (2 ~6 years, n=484)	BPb, EPb	Elevated HCT (p<0.01), monocytes (p<0.05) and decrease MCHC (p<0.01) in Guiyu children. BPb negatively correlated with the HCT, MCV, Hgb and MCH (all p<0.01), positively associated with RDW (p<0.05). EPb also negatively correlated with the HCT, MCV, Hgb and MCH (all p<0.001), positively associated with RDW (p<0.01).
(Zheng et al., 2019)	Guiyu vs. Haojiang (3 ~7 years, n=203)	BPb	Elevated monocytes, neutrophils and leukocytes (all p<0.01) in Guiyu children. Pb positively related to neutrophil and monocyte.
(Chen et al., 2019)	Guiyu vs. Jinping (4~85 years, n=267)	BPb	Higher Hgb (137.0 vs. 123.0 g/L) and RBC (both p=0.001). Pb positively correlated with Hgb.
(Sun et al., 2012)	Guiyu vs. Haojiang (3 ~6 years, n=237)	BPb	Elevated eosinophils and neutrophil.
(Chen et al., 2021)	Guiyu vs. Haojiang (3 ~7 years, n=486)	BPb	Elevated blood Pb levels were significantly associated with higher counts of WBCs and neutrophils.
(Wang et al., 2021)	Guiyu vs. Haojiang (3 ~6 years, n=222)	BPb	Hgb level decreased with elevate BPb. Prevalence of anaemia significantly higher in the exposed group.
(Zhang et al., 2020a)	Guiyu vs. Haojiang (3 ~7 years, n=207)	BPb	BPb positively correlated with peripheral monocyte percentage.
**Growth effects**			
(Zheng et al., 2008)	Guiyu vs. Chendian (1~7 years, n=278)	BPb	Lower height (104.35 vs. 105.81 cm, p<0.01). Significant increasing trend in Pb with increasing age in Guiyu (p<0.01).
(Zhang et al., 2017a)	Guiyu vs. Haojiang (4~ 7 years, n=118)	BPb	Lower height (107.62 vs. 112.53, p<0.01), weight (17.47 vs. 18.72, p<0.05) and chest circumference (50.56 vs. 51.80 cm, p<0.05), no differences in head circumference.
(Zeng et al., 2017)	Guiyu vs. Haojiang & Xiashan (5 ~7 years, n=206)	BPb	Lower height (111.03 vs. 112.56 cm, p<0.05), no differences in weight and BMI.
(Yang et al., 2013)	Guiyu only (3 ~8 years, n=246)	BPb	Pb negatively correlated with both height and weight, positively correlated with bone resorption biomarker.

(Xu et al., 2014)	Guiyu only (4 ~6 years, n=162)	BPb	Pb negatively correlated with weight.
(Huo et al., 2007)	Guiyu vs. Chendian (median 5.0 years, n=226)	BPb	No differences in height, weight, chest circumference, or head circumference.
(Lin et al., 2017b)	Guiyu vs. Haojiang (3 ~7 years, n=284)	BPb	Lower weight (17.05 vs. 18.00 kg, $p<0.05$), chest circumference (51.51 vs. 52.81 cm, $p<0.01$) and head circumference (49.82 vs. 50.47 cm, $p=0.05$), no difference in height.
(Zeng et al., 2019a)	Guiyu vs. Haojiang (preschool, n=470)	BPb	Lower chest circumference (51.18 vs. 51.95 cm, $p<0.05$) and BMI (15.61 vs. 16.12 kg/m^2, $p<0.01$). Pb negatively correlated with height ($p<0.001$), weight ($p<0.001$), BMI ($p<0.05$), head circumference ($p<0.05$), and chest circumference ($p<0.05$).
(Lu et al., 2018)	Guiyu vs. Haojiang (3 ~7 years, n=590)	BPb	Higher BMI (15.67 vs. 15.06 kg/m^2, $p<0.001$).
(Zeng et al., 2018)	Guiyu vs. Haojiang (3 ~7 years, n=466)	BPb	No differences in height, weight or BMI.
(Cao et al., 2018)	Guiyu vs. Haojiang (3 ~7 years, n=118)	BPb	No differences in height, weight.
(Zhang et al., 2016)	Guiyu vs. Haojiang (3 ~7 years, n=411)	BPb	No differences in height, weight or BMI.
(Xu et al., 2020)	Guiyu vs. Haojiang (3 ~7 years, n=116)	BPb	No differences in height, weight or BMI.
(Xu et al., 2015c)	Guiyu vs. Chendian (3 ~7 years, n=167)	BPb	Higher BMI (15.3 vs. 14.8 kg/m^2, $p<0.01$). No differences in height, weight, chest circumference or head circumference (all $p>0.05$). Pb positively correlated with height.
(Zhang et al., 2017b)	Guiyu vs. Haojiang (3 ~7 years, n=294)	BPb	Lower BMI (14.73 vs. 16.06 kg/m^2, $p<0.001$).

(Lin et al., 2016)	Guiyu vs. Haojiang (2 ~7 years, n=378)	BPb	No differences in height, weight, chest circumference or head circumference.
(Zeng et al., 2016)	Guiyu vs. Haojiang (3 ~8 years, n=470)	BPb	Lower chest circumference (51.18 vs. 51.95 cm, p<0.05) and BMI (15.61 vs. 16.12 kg/m^2, p<0.01) in Guiyu children, no differences in head circumference, height or weight.
(Liu et al., 2018a)	Guiyu vs. Nanao (3 years, n=284)	BPb	Higher BMI (15.47 vs. 14.96 kg/m^2, p<0.01).
(Guo et al., 2014)	Guiyu vs. Haojiang (under 11 years, n=837)	BPb	Lower height (105.95 vs. 104.52) and weight (17.25 vs. 17.72 kg) (both p<0.05), no differences in chest circumference, head circumference (both p>0.05).
(Xu et al., 2015a)	Guiyu vs. Haojiang (3 ~7 years, n=590)	BPb	Lower weight (17.14 vs. 17.94 kg) and chest circumference (51.65 vs. 52.53 cm) (both p<0.01), no differences in height and head circumference.
(Zheng et al., 2019)	Guiyu vs. Haojiang (3 ~7 years, n=203)	BPb	Lower height (104.70 vs. 108.71), weight (16.68 vs. 18.81 kg) and BMI (15.17 vs. 15.80 kg/m^2) (all p<0.01).
(Liu et al., 2015b)	Guiyu vs. Nanao (3 years, n=284)	BPb	Higher BMI (15.47 vs. 14.96 kg/m^2, p<0.01).
(Zheng et al., 2021)	Guiyu vs. Haojiang (2 ~7 years, n=324)	BPb	Higher BMI (15.57 vs. 15.08 kg/m^2, p=0.025).
(Chen et al., 2021)	Guiyu vs. Haojiang (3 ~7 years, n=486)	BPb	Lower BMI (14.7 vs. 15.9 kg/m^2, p<0.001)
(Hou et al., 2020)	Guiyu vs. Haojiang	BPb	Lower BMI (14.97 vs. 15.42 kg/m^2, p<0.001)
(Zhang et al., 2020b)	Guiyu vs. Haojiang (2 ~7 years, n=147)	BPb	No differences in BMI.
(Zhang et al., 2020a)	Guiyu vs. Haojiang (3 ~7 years, n=207)	BPb	No differences in BMI.
**Adverse birth outcomes**			

(Xu et al., 2012)	Guiyu vs. Xiamen (Neonate, n=244493)	UCB-Pb	Higher rates of adverse birth outcomes (stillbirth, low birth weight, term low birth weight and lower Apgar scores) (all p<0.01). birth weight 3.17 kg vs. 3.26 kg, p<0.01
(Li et al., 2008c)	Guiyu vs. Chaonan (Neonate, n=152)	MPb, CBPb	No differences in birth height, birth weight, or Apgar scores.
(Lin et al., 2013)	Guiyu vs. Chaonan (mother–infant pairs, n=320)	PCPb	Lower birth weight (p<0.05) and Apgar scores (p<0.01), longer birth length (p<0.05).
(Guo et al., 2010)	Guiyu vs. Shantou (mother–infant pairs, n=220)	PCPb	No differences in birth height, birth weight, or Apgar scores.
(Zhang et al., 2011)	Guiyu vs. Shantou (mother–infant pairs, n=105)	PCPb	No differences in birth height, birth weight, or Apgar scores.
(Qiu, 2012)	Guiyu vs. Control (mother-infant pairs, n=281)	PCPb	Shorter birth length (p<0.01). No difference in birth weight.
(Xu et al., 2016)	Guiyu vs. Haojiang (mother–infant pairs, n=185)	PCPb	Shorter birth length in Guiyu neonates (p<0.01)
(Ni et al., 2014b)	Guiyu vs. Shantou (mother-infant pairs, n=201)	UCB-Pb	No differences in birth length, birth weight.
(Zeng et al., 2016)	Guiyu vs. Haojiang (3 ~8 years, n=470)	BPb	Lower birth weight (p<0.01), shorter birth length (p<0.05).
(Liu et al., 2018a)	Guiyu vs. Nanao (3 years, n=284)	BPb	No differences in birth BMI.

(Zeng et al., 2019a)	Guiyu vs. Haojiang (preschool, n=470)	BPb	Lower birth weight (p<0.01), shorter birth length (p<0.05).
(Zeng et al., 2017)	Guiyu vs. Haojiang & Xiashan (5 ~7 years, n=206)	BPb	Lower birth weight (p<0.05).
(Huo et al., 2014)	Guiyu vs. Chendian (mother-infant pairs, n=504)	BPb	No differences in birth height, birth weight, or Apgar scores.
(Kim et al., 2020)	Guiyu vs. Shantou (mother-infant pairs, n=634)	BPb	Lower birth weight, head circumference and BMI, longer birth length
(Zeng et al., 2019b)	Guiyu vs. Haojiang (mother-infant pairs, n=204)	UCB-Pb	Lower birth BMI (p<0.001).
**Respiratory system**			
(Zeng et al., 2017)	Guiyu vs. Haojiang & Xiashan (5 ~7 years, n=206)	BPb	Lower lung function (FVC and FEV1) levels in Guiyu children. No significant difference in FVC/FEV1. No significant association between Pb and FVC, FEV1.
(Zeng et al., 2016)	Guiyu vs. Haojiang (3 ~8 years, n=470)	BPb	Higher prevalence of cough, phlegm, dyspnea, and wheeze were in Guiyu children.
**Thyroid function**			
(Liu et al., 2018a)	Guiyu vs. Nanao (3 years, n=284)	BPb	Higher levels of FT4 (16.65 vs. 16.06 pmol/L, p<0.01) and TSH (2.79 vs. 2.21 mIU/L, p<0.01) in Guiyu children. Exposure to Pb affects thyroid function. Both FT4 and TSH were positively related to the recycling industry.
**Hepatic function**			

(Chen et al., 2019)	Guiyu vs. Jinping (4~85 years, n=267)	BPb	Higher activity of serum GGT (p<0.005) in Guiyu residents. Exposure of Pb is linked with risk of abnormal liver function.
**Oral anti-inflammatory and anti-microbial ability**			
(Hou et al., 2020)	Guiyu vs. Haojiang (2.5 ~6 years, n=574)	BPb	BPb negatively correlated with salivary sialic acids. The prevalence of dental caries in deciduous teeth was significantly higher in the Guiyu children than in Haojiang.
(Zhang et al., 2020a)	Guiyu vs. Haojiang (3 ~7 years, n=207)	BPb	BPb negatively correlated with saliva salivary agglutinin (SAG).
**Gestational age (weeks)**			
(Xu et al., 2016)	Guiyu vs. Haojiang (mother–infant pairs, n=185)	PCPb	Longer gestational age (40.86 vs. 39.54, p<0.001) in Guiyu group. No significant correlation between Pb and gestational age.
(Xu et al., 2012)	Guiyu vs. Xiamen (Neonate, n= 244493)	UCB-Pb	Longer gestational age (39.5 vs. 38.9, p<0.01).
(Li et al., 2008c)	Guiyu vs. Chaonan (Neonate)	MPb, UCB-Pb	No significant difference of gestational age.
(Guo et al., 2010)	Guiyu vs. Shantou (mother–infant pairs, n=220)	PCPb	No significant difference of gestational age.
(Lin et al., 2013)	Guiyu vs. Chaonan (mother–infant pairs, n=320)	PCPb	Longer gestational age (39.69 vs. 39.28, p<0.05).
(Zhang et al., 2011)	Guiyu vs. Shantou (mother–infant pairs, n=105)	PCPb	No significant difference of gestational age.
(Qiu et al., 2012)	Guiyu vs. Shantou	PCPb	PCPb correlated with gestational age.

	(mother–infant pairs, n=251)		
(Huo et al., 2014)	Guiyu vs. Chendian (mother–infant pairs, n=504)	UCB-Pb	No significant difference of gestational age between two groups.
**Chromosome, DNA damage and epigenotoxic**			
(Ni et al., 2014b)	Guiyu vs. Shantou (pregnant women, n=201)	UCB-Pb	No significant association between 8-OHdG and CBPb.
(Lin et al., 2013)	Guiyu vs. Chaonan (Neonate, n=320)	PCPb	No significant correlation between PCPb and placental telomere length.
(Zhang et al., 2011)	Guiyu vs. Shantou (mother–infant pairs, n=105)	PCPb	Higher MT levels ($p<0.01$), and lower S100P protein levels ($p<0.05$) and mRNA ($p<0.001$) in Guiyu placentas. No correlation between PCPb and S100P or MT expression.
(Xu et al., 2018)	Guiyu only (3 ~6 years, n=118)	BPb	8-OHdG 407.79 (152.05~876.26) ng/g creatinine, hOGG1 mRNA expression level 0.038 (0.009~0.542). Children with high Pb had significantly higher 8-OHdG than low Pb exposure.
(Liao et al., 2011)	Guiyu only (3 ~7 years, n=118)	BPb	Pb exposure can affect urine 8-OHdG level, association between increase of oxidative stress level and higher Pb.
(Zeng et al., 2019b)	Guiyu vs. Haojiang (mother-infant pairs, n=204)	UCB-Pb	Differential methylation of two CpGs of BAI1 and CTNNA2 involved in brain neuronal development in newborns which were associated with the maternal Pb exposure

* blood Pb (BPb); erythrocyte Pb (EPb); Umbilical cord blood Pb (UCB-Pb); placenta Pb (PCPb); meconium Pb (MPb);

Reference:

Cai, H., Xu, X., Zhang, Y., Cong, X., Lu, X., Huo, X., 2019. Elevated lead levels from e-waste exposure are linked to sensory integration difficulties in preschool children. Neurotoxicology 71, 150-158.

Cao, J., Xu, X., Zhang, Y., Zeng, Z., Hylkema, M.N., Huo, X., 2018. Increased memory T cell populations in Pb-exposed children from an e-waste-recycling area. Sci Total Environ 616-617, 988-995.

Chen, X., Zhang, R., Peng, Z., Chen, X., Chen, L., Wang, H., Cai, Z., Qin, S., Liu, B., Huo, X., Lin, W., Wang, T., Ma, L., 2013. Effects of lead exposure on attention deficit hyperactivity disorder in children from an e-waste recycling area. Beijing Medical Journal (*in Chinese*) 35, 249-252.

Chen, Y., Xu, X., Zeng, Z., Lin, X., Qin, Q., Huo, X., 2019. Blood lead and cadmium levels associated with hematological and hepatic functions in patients from an e-waste-polluted area. Chemosphere 220, 531-538.

Chen, Z., Huo, X., Zhang, S., Cheng, Z., Huang, Y., Xu, X., 2021. Relations of blood lead levels to echocardiographic left ventricular structure and function in preschool children. Chemosphere 268, 128793.

Dai, Y., Huo, X., Zhang, Y., Yang, T., Li, M., Xu, X., 2017. Elevated lead levels and changes in blood morphology and erythrocyte CR1 in preschool children from an e-waste area. Sci Total Environ 592, 51-59.

Guo, P., Xu, X., Huang, B., Sun, D., Zhang, J., Chen, X., Zhang, Q., Huo, X., Hao, Y., 2014. Blood lead levels and associated factors among children in Guiyu of China: a population-based study. PLoS One 9, e105470.

Guo, Y., Huo, X., Li, Y., Wu, K., Liu, J., Huang, J., Zheng, G., Xiao, Q., Yang, H., Wang, Y., Chen, A., Xu, X., 2010. Monitoring of lead, cadmium, chromium and nickel in placenta from an e-waste recycling town in China. Sci Total Environ 408, 3113-3117.

Han, D., Huo, X., Zheng, L., Li, Y., 2007. Investigation on blood lead and intelligence levels of children in electronic waste recycling area. Journal of Shantou University Medical College (*in Chinese*), 170-172+175.

Hou, R., Huo, X., Zhang, S., Xu, C., Huang, Y., Xu, X., 2020. Elevated levels of lead exposure and impact on the anti-inflammatory ability of oral sialic acids among preschool children in e-waste areas. Sci Total Environ 699, 134380.

Huang, Y., Ni, W., Chen, Y., Wang, X., Zhang, J., Wu, K., 2015. Levels and risk factors of antimony contamination in human hair from an electronic waste recycling area, Guiyu, China. Environ Sci Pollut Res Int 22, 7112-7119.

Huo, X., Dai, Y., Yang, T., Zhang, Y., Li, M., Xu, X., 2019. Decreased erythrocyte CD44 and CD58 expression link e-waste Pb toxicity to changes in erythrocyte immunity in preschool children. Sci Total Environ 664, 690-697.

Huo, X., Peng, L., Qiu, B., Zheng, L., Yekeen, T.A., Xu, X., 2014. ALAD genotypes and blood lead levels of neonates and children from e-waste exposure in Guiyu, China. Environ Sci Pollut Res Int 21, 6744-6750.

Huo, X., Peng, L., Xu, X., Zheng, L., Qiu, B., Qi, Z., Zhang, B., Han, D., Piao, Z., 2007. Elevated blood lead levels of children in Guiyu, an electronic waste recycling town in China. Environ Health Perspect 115, 1113-1117.

Kim, S., Xu, X., Zhang, Y., Zheng, X., Liu, R., Dietrich, K., Reponen, T., Ho, S.M., Xie, C., Sucharew, H., Huo, X., Chen, A., 2019. Metal concentrations in pregnant women and neonates from informal electronic waste recycling. J Expo Sci Environ Epidemiol 29, 406-415.

Kim, S.S., Xu, X., Zhang, Y., Zheng, X., Liu, R., Dietrich, K.N., Reponen, T., Xie, C., Sucharew, H., Huo, X., Chen, A., 2020. Birth outcomes associated with maternal exposure to metals from informal electronic waste recycling in Guiyu, China. Environ Int 137, 105580.
Li, Y., Huo, X., Liu, J., Peng, L., Li, W., Xu, X., 2011. Assessment of cadmium exposure for neonates in Guiyu, an electronic waste pollution site of China. Environ Monit Assess 177, 343-351.
Li, Y., Huo, X., Wu, K., Liu, J., Ban, H., Xu, X., 2010. Placental cadmium concentration and the levels of metallothionein expression in an electronic waste environmental polluted site. Carcinogenesis,Teratogenesis & Mutagenesis (*in Chinese*) 22, 115-118.
Li, Y., Xu, X., Liu, J., Wu, K., Gu, C., Shao, G., Chen, S., Chen, G., Huo, X., 2008a. The hazard of chromium exposure to neonates in Guiyu of China. Sci Total Environ 403, 99-104.
Li, Y., Xu, X., Liu, J., Zheng, G., Chen, S., Huo, X., 2008b. Determination of meconium lead Level of newborn by graphite furnace atomic absorption spectrometry. Spectroscopy and Spectral Analysis (in Chinese), 447-449.
Li, Y., Xu, X., Wu, K., Chen, G., Liu, J., Chen, S., Gu, C., Zhang, B., Zheng, L., Zheng, M., Huo, X., 2008c. Monitoring of lead load and its effect on neonatal behavioral neurological assessment scores in Guiyu, an electronic waste recycling town in China. J Environ Monit 10, 1233-1238.
Liao, W., Huo, X., Liu, W., Yang, H., Ji, M., Xu, X., 2011. Influence of lead exposure on urine 8-hydroxy-2'-deoxyguanosine of children in an electronic waste recycling area. Journal of Shantou University Medical College (*in Chinese*) 24, 32-34.
Lin, J., Huo, X., Huang, J., Zheng, G., Xu, X., 2010. Relationship between blood cadmium and red blood cell parameters in children aged 3~7 years in Guiyu area of Guangdong Province. Journal of Shantou University Medical College (*in Chinese*) 23, 100-101+110.
Lin, S., Huo, X., Zhang, Q., Fan, X., Du, L., Xu, X., Qiu, S., Zhang, Y., Wang, Y., Gu, J., 2013. Short placental telomere was associated with cadmium pollution in an electronic waste recycling town in China. PLoS One 8, e60815.
Lin, X., Du, D., Chen, S., Chen, X., 2017a. The correlation between heavy metal exposure and parkinson disease in electronic waste dismantling area. Shenzhen Journal of Integrated Traditional Chinese and Western Medicine November (*in Chinese*) 27, 1-3.
Lin, X., Xu, X., Zeng, X., Xu, L., Zeng, Z., Huo, X., 2017b. Decreased vaccine antibody titers following exposure to multiple metals and metalloids in e-waste-exposed preschool children. Environ Pollut 220, 354-363.
Lin, Y., Xu, X., Dai, Y., Zhang, Y., Li, W., Huo, X., 2016. Considerable decrease of antibody titers against measles, mumps, and rubella in preschool children from an e-waste recycling area. Sci Total Environ 573, 760-766.
Liu, C., Huo, X., Lin, P., Zhang, Y., Li, W., Xu, X., 2015a. Association between blood erythrocyte lead concentrations and hemoglobin levels in preschool children. Environ Sci Pollut Res Int 22, 9233-9240.
Liu, J., Xu, X., Wu, K., Piao, Z., Huang, J., Guo, Y., Li, W., Zhang, Y., Chen, A., Huo, X., 2011a. Association between lead exposure from electronic waste recycling and child temperament alterations. Neurotoxicology 32, 458-464.
Liu, L., Xu, X., Yekeen, T.A., Lin, K., Li, W., Huo, X., 2015b. Assessment of association between the dopamine D2 receptor (DRD2) polymorphism and neurodevelopment of children exposed to lead. Environ Sci Pollut Res Int 22, 1786-1793.
Liu, L., Zhang, B., Lin, K., Zhang, Y., Xu, X., Huo, X., 2018a. Thyroid disruption and reduced mental development in children from an informal e-waste recycling area: A mediation analysis. Chemosphere

193, 498-505.
Liu, W., Huo, X., Liu, D., Zeng, X., Zhang, Y., Xu, X., 2014. S100β in heavy metal-related child attention-deficit hyperactivity disorder in an informal e-waste recycling area. Neurotoxicology 45, 185-191.
Liu, W., Yang, H., Liao, W., Qiu, S., Huo, X., Xu, X., 2011b. Study on blood chromium levels and behavior effect of children in electronic waste recycling area. Journal of Shantou University Medical College (*in Chinese*) 24, 26-29.
Liu, Y., Huo, X., Xu, L., Wei, X., Wu, W., Wu, X., Xu, X., 2018b. Hearing loss in children with e-waste lead and cadmium exposure. Sci Total Environ 624, 621-627.
Lu, X., Xu, X., Zhang, Y., Zhang, Y., Wang, C., Huo, X., 2018. Elevated inflammatory Lp-PLA2 and IL-6 link e-waste Pb toxicity to cardiovascular risk factors in preschool children. Environ Pollut 234, 601-609.
Ni, W., Chen, Y., Huang, Y., Wang, X., Zhang, G., Luo, J., Wu, K., 2014a. Hair mercury concentrations and associated factors in an electronic waste recycling area, Guiyu, China. Environ Res 128, 84-91.
Ni, W., Huang, Y., Wang, X., Zhang, J., Wu, K., 2014b. Associations of neonatal lead, cadmium, chromium and nickel co-exposure with DNA oxidative damage in an electronic waste recycling town. Sci Total Environ 472, 354-362.
Peng, L., Huo, X., Xu, X., Zhang, Y., Qiu, B., 2005. Effects of electronic waste recycling-disposing contamination on children's blood lead level. Journal of Shantou University Medical College (*in Chinese*), 48-50.
Qiu, S., 2012. Lead and Cadmium Occurrence in Placenta and Associated Factors in Guiyu, China. 2012.
Qiu, S., Huo, X., Liu, J., Li, B., Akangbe, Y.T., Zhang, Y., Xu, X., 2012. Association between prenatal lead and cadmium exposure from electronic waste recycling and the development of neonate. Journal of Shantou University Medical College (*in Chinese*) 25, 138-140.
Sun, D., Zhang, J., Huang, B., Zhang, Y., Xu, X., Huo, X., 2012. Effect of lead and cadmium exposure on the levels of innate immune cell in preschool children. Journal of Shantou University Medical College (*in Chinese*) 25, 193-195.
Wang, H., Huang, P., Zhang, R., Feng, X., Tang, Q., Liu, S., Wen, F., Zeng, L., Liu, Y., Wang, T., Ma, L., 2021. Effect of lead exposure from electronic waste on haemoglobin synthesis in children. Int Arch Occup Environ Health.
Xu, L., Ge, J., Huo, X., Zhang, Y., Lau, A.T.Y., Xu, X., 2016. Differential proteomic expression of human placenta and fetal development following e-waste lead and cadmium exposure in utero. Sci Total Environ 550, 1163-1170.
Xu, L., Huo, X., Liu, Y., Zhang, Y., Qin, Q., Xu, X., 2020. Hearing loss risk and DNA methylation signatures in preschool children following lead and cadmium exposure from an electronic waste recycling area. Chemosphere 246, 125829.
Xu, X., Chen, X., Zhang, J., Guo, P., Fu, T., Dai, Y., Lin, S.L., Huo, X., 2015a. Decreased blood hepatitis B surface antibody levels linked to e-waste lead exposure in preschool children. J Hazard Mater 298, 122-128.
Xu, X., Chiung, Y.M., Lu, F., Qiu, S., Ji, M., Huo, X., 2015b. Associations of cadmium, bisphenol A and polychlorinated biphenyl co-exposure in utero with placental gene expression and neonatal outcomes. Reprod Toxicol 52, 62-70.
Xu, X., Liao, W., Lin, Y., Dai, Y., Shi, Z., Huo, X., 2018. Blood concentrations of lead, cadmium, mercury

and their association with biomarkers of DNA oxidative damage in preschool children living in an e-waste recycling area. Environ Geochem Health 40, 1481-1494.
Xu, X., Liu, J., Huang, C., Lu, F., Chiung, Y.M., Huo, X., 2015c. Association of polycyclic aromatic hydrocarbons (PAHs) and lead co-exposure with child physical growth and development in an e-waste recycling town. Chemosphere 139, 295-302.
Xu, X., Liu, J., Zeng, X., Lu, F., Chen, A., Huo, X., 2014. Elevated serum polybrominated diphenyl ethers and alteration of thyroid hormones in children from Guiyu, China. PLoS One 9, e113699.
Xu, X., Peng, L., Li, W., Qiu, B., Huo, X., 2006. Investigation of blood lead level of children aged 1~6 in electronic waste disposing district. Journal of Environment and Health (*in Chinese*), 58-60.
Xu, X., Yang, H., Chen, A., Zhou, Y., Wu, K., Liu, J., Zhang, Y., Huo, X., 2012. Birth outcomes related to informal e-waste recycling in Guiyu, China. Reprod Toxicol 33, 94-98.
Xu, X., Yekeen, T.A., Liu, J., Zhuang, B., Li, W., Huo, X., 2015d. Chromium exposure among children from an electronic waste recycling town of China. Environ Sci Pollut Res Int 22, 1778-1785.
Yang, H., Huo, X., Yekeen, T.A., Zheng, Q., Zheng, M., Xu, X., 2013. Effects of lead and cadmium exposure from electronic waste on child physical growth. Environ Sci Pollut Res Int 20, 4441-4447.
Zeng, X., Xu, C., Xu, X., Zhang, Y., Huang, Y., Huo, X., 2021. Elevated lead levels in relation to low serum neuropeptide Y and adverse behavioral effects in preschool children with e-waste exposure. Chemosphere 269, 129380.
Zeng, X., Xu, X., Boezen, H.M., Vonk, J.M., Wu, W., Huo, X., 2017. Decreased lung function with mediation of blood parameters linked to e-waste lead and cadmium exposure in preschool children. Environ Pollut 230, 838-848.
Zeng, X., Xu, X., Qin, Q., Ye, K., Wu, W., Huo, X., 2019a. Heavy metal exposure has adverse effects on the growth and development of preschool children. Environ Geochem Health 41, 309-321.
Zeng, X., Xu, X., Zheng, X., Reponen, T., Chen, A., Huo, X., 2016. Heavy metals in PM2.5 and in blood, and children's respiratory symptoms and asthma from an e-waste recycling area. Environ Pollut 210, 346-353.
Zeng, Z., Huo, X., Zhang, Y., Hylkema, M.N., Wu, Y., Xu, X., 2019b. Differential DNA methylation in newborns with maternal exposure to heavy metals from an e-waste recycling area. Environ Res 171, 536-545.
Zeng, Z., Huo, X., Zhang, Y., Xiao, Z., Zhang, Y., Xu, X., 2018. Lead exposure is associated with risk of impaired coagulation in preschool children from an e-waste recycling area. Environ Sci Pollut Res Int 25, 20670-20679.
Zhang, B., Huo, X., Xu, L., Cheng, Z., Cong, X., Lu, X., Xu, X., 2017a. Elevated lead levels from e-waste exposure are linked to decreased olfactory memory in children. Environ Pollut 231, 1112-1121.
Zhang, Q., Zhou, T., Xu, X., Guo, Y., Zhao, Z., Zhu, M., Li, W., Yi, D., Huo, X., 2011. Downregulation of placental S100P is associated with cadmium exposure in Guiyu, an e-waste recycling town in China. Sci Total Environ 410-411, 53-58.
Zhang, R., Huo, X., Ho, G., Chen, X., Wang, H., Wang, T., Ma, L., 2015. Attention-deficit/hyperactivity symptoms in preschool children from an e-waste recycling town: assessment by the parent report derived from DSM-IV. BMC Pediatr 15, 51.
Zhang, S., Huo, X., Li, M., Hou, R., Cong, X., Xu, X., 2020a. Oral antimicrobial activity weakened in children with electronic waste lead exposure. Environ Sci Pollut Res Int 27, 14763-14770.
Zhang, Y., Huo, X., Cao, J., Yang, T., Xu, L., Xu, X., 2016. Elevated lead levels and adverse effects on natural killer cells in children from an electronic waste recycling area. Environ Pollut 213, 143-150.

Zhang, Y., Huo, X., Lu, X., Zeng, Z., Faas, M.M., Xu, X., 2020b. Exposure to multiple heavy metals associate with aberrant immune homeostasis and inflammatory activation in preschool children. Chemosphere 257, 127257.

Zhang, Y., Xu, X., Chen, A., Davuljigari, C.B., Zheng, X., Kim, S.S., Dietrich, K.N., Ho, S.M., Reponen, T., Huo, X., 2018. Maternal urinary cadmium levels during pregnancy associated with risk of sex-dependent birth outcomes from an e-waste pollution site in China. Reprod Toxicol 75, 49-55.

Zhang, Y., Xu, X., Sun, D., Cao, J., Zhang, Y., Huo, X., 2017b. Alteration of the number and percentage of innate immune cells in preschool children from an e-waste recycling area. Ecotoxicol Environ Saf 145, 615-622.

Zheng, G., Xu, X., Li, B., Wu, K., Yekeen, T.A., Huo, X., 2013. Association between lung function in school children and exposure to three transition metals from an e-waste recycling area. J Expo Sci Environ Epidemiol 23, 67-72.

Zheng, L., Wu, K., Li, Y., Qi, Z., Han, D., Zhang, B., Gu, C., Chen, G., Liu, J., Chen, S., Xu, X., Huo, X., 2008. Blood lead and cadmium levels and relevant factors among children from an e-waste recycling town in China. Environ Res 108, 15-20.

Zheng, X., Huo, X., Zhang, Y., Wang, Q., Zhang, Y., Xu, X., 2019. Cardiovascular endothelial inflammation by chronic coexposure to lead (Pb) and polycyclic aromatic hydrocarbons from preschool children in an e-waste recycling area. Environ Pollut 246, 587-596.

Zheng, X., Xu, X., Lu, F., Wang, Q., Zeng, Z., Huo, X., 2021. High serum IgG subclass concentrations in children with e-waste Pb and Cd exposure. Sci Total Environ 764, 142806.

Zhuang, B., Lu, F., Fu, T., Liu, Y., Huo, X., Xu, X., 2015. Investigation and analysis of prenatal mercury exposure in neonates. Journal of Shantou University Medical College (*in Chinese*) 28, 10-12.
